# The consequence of the credit risk on the financial performance in light of COVID-19: Evidence from Islamic versus conventional banks across MEA region

**DOI:** 10.1186/s43093-022-00122-y

**Published:** 2022-07-22

**Authors:** Hussien Mohsen Ahmed, Sherif Ismail El-Halaby, Hebatallah Ahmed Soliman

**Affiliations:** 1 Faculty of Commerce English Section, Helwan University, Helwan, Egypt; 2 Faculty of management sciences, MSA University, 6th of October City, Egypt; 3 Faculty of Business Administration, Sinai University, Ismailia, Egypt

**Keywords:** COVID-19 pandemic, Credit risk, Financial performance, Islamic, Convectional banks, MEA region

## Abstract

**Purpose:**

The increased number of nonperforming loans (NPLs) during COVID-19 pandemic has interrogated the robustness of banks and stability of the whole banking segment. We examine the impact of credit risk (CR) on financial performance (FP) by comparing Islamic banks (IBs) to conventional banks (CBs). We also investigate the influence of COVID-19 on this association.

**Design/methodology/approach:**

Our sample includes the largest 200 banks across 15 countries from the Middle East and the Africa (MEA) region over a four-year period (2018–2021). Panel ordinary least squares (OLS) with fixed and random effects were used.

**Findings:**

We find a negative association between NPLs and FP for IBs and CBs. We reveal that COVID-19 is partially mediated the association between NPLs and FP in case of the whole sample and separated sample of CBs while not in case of IBs.

**Originality:**

The evidence of CR and FP on samples of financial sector across MEA region has not been studied in the era of COVID-19 as far as we know.

**Research limitations/implications:**

This study contributes to the knowledge of the risk and financial performance during the crisis nexus and provides information that is valued to bankers, academics, managers and regulators for policy formulation.

## Introduction

The coronavirus is a philanthropic crisis, which continues to affect survives and livelihoods in the whole world. It has enforced national and regional economies to close for months and years at a time, causing adversity for the global world. At the time of writing by June 30, 2021, the coronavirus outbreak has feast across the world and has led to more than 186,411,011 million confirmed cases and 4,031,725 million deaths [105]. The COVID-19 has led to rehabilitated interest in communicable disease surveillance, control and in the economic influence of such diseases.

From the perception of financial institutions as banks, COVID-19 activated has definite consequences for managing and mitigating the credit risk (CR). The banks have been modifying to the original dynamics and sightseeing possible novel methods to the challenges. The investigations assess the impact of the crisis on economies, the impact by sector, and specific CR issues requiring real-time monitoring. Crises like COVID -19 and financial global crisis are one of the most instantaneous and significant issues for the banking sector, particularly in emerging countries. The latest COVID -19 pandemic subsequent in financial market instability, which upsurge the strictness of its impact, was that it occurred at a time when the whole world generally and emerging region particularly like the Middle East and North Africa (MENA), Gulf Cooperation Council (GCC) and African countries did not recover as required from the financial crisis.

Banks face numerous risks as liquidity, credit, operational, foreign exchange, as well as market risks. Cornett and Saunders [26] classify these risks into three clusters, namely operational, financial and strategic. Hussain and Al-Ajmi [44] claim that CR is one of the greatest challenging risks faced by banks. CR is defined as the likelihood of a loss subsequent from the debtor's failure to pay the predefined obligation [20]. However, bank managers add to bank default if they fail to efficiently assess the solvency of the debtors. Evaluating CR and creating appropriate allowances for doubtful debts may support banks to circumvent this kind of risk. However, very often when banks extend credit for investors, no payment is predictable on the loan from the debtors. This can eventually put a strain on FP, which could lead to the letdown of the bank. Therefore, after the financial crisis, the risk management in the majority of banks globally is frequently focused on CR [65].

The nonperforming loans (NPLs) after the global financial crises based on Ghosh [37] are essentially becoming under the eyes of government and banking management since they are considered with the failure of banking system. For Kargi [53], banks may rise their income, thus satisfying their objective through extending huge amounts of credit. But, when they could not collect these loans, the profitability will droplet. The previous literature displays that CR and FP are inversely associated [93]. Conversely, upper risk yields upper profit and the two are straight proportional to each other [77].

Ivanovic [47] argues that NPLs can be used as a signal of banking crises as it affects the economic development of country through lessening the credit development. A low degree of NPLs displays a robust monetary system of the country, whereas great NPLs specify a feeble financial position. The cumulative level of NPLs for Souza and Feijo [99] will affect the banks in the long run and then affect the financial position of the country’ economy. According to Vouldis and Louzis [103], the growing drift of NPLs will affect the efficiency of banking system, which results in banking crises. Michael et al. [71] specified that NPLs affect the whole routine of the banks, therefore intimidating the FP and reputation of the banking sector. The level of NPLs affects directly on the banks’ FP. NPLs for Samir and Kamra [95] have a negative influence on FP as they reduce the interest earnings and erode the existing incomes and capital base by allowances. Biabani et al. [15] argue that when NPLs exceed bank capital in an important number of banks, a bank crisis develops, that ultimately leads to financial crisis. NPLs will block the interest income, then decrease the investment beginnings and develop liquidity crises in the financial system, which results in sinking level of FP then insolvency problematic. Therefore, it is crucial to recognize and measure the consequence of NPLs on FP during the current pandemic in a new context.

CR in Islamic banks (IBs), and how it compares with conventional banks (CBs), has been discussed in Chamberlain et al. [23]. However, the outcomes of these studies are mixed and questionable. More investigation including original data, measurement rules and estimation methods is requisite. This is the task of our present study, which focusses on the banking industry in MEA region and, in doing so, complements other exertions to measure and explain the influence of CR on FP in IBs relative to CBs during the COVID-19. There is a fast-mounting body of literature on the influence of COVID-19 over the economy and financial markets [3]. Closely associated are numerous latest studies focusing on the impression of COVID-19 on CR (e.g., [54]). Similarly, there are several studies measuring the effects of COVID-19 or other crisis on FP [92]. It is becoming gradually seeming that NPLs are probably to become one of the utmost thoughtful significances of the pandemic and it is impact over the performance. This paper focuses on the effect of COVID-19 but as a mediator for the association between FP and CR.

This study is differing from the other literature in different aspects. While Mushafiq et al. [77] study the association between CR and FP in non-financial corporations, we focus on financial sector. Whereas Hunjra et al. [43] used data for only CBs, our sample used data for IBs and CBs. Whereas Agyapong [5] employed PLS-SEM technique to analyze financial risks and its impact on the Small- and Mid-size Enterprise (SMEs), we employed regression analysis for banks. While most of the literature measured the NPLs on one country (e.g., Mushafiq et al. [77], Ekinci and Poyraz [31], our study measures NPLs across countries. While Akram and Rahman [7] compare CR management of IBs and CBs in Pakistan, we extend this research by measuring the impact of CR on FP with considering the impact of pandemic. Similarly, while we consider the impact of COVID-19 on the behavior of IBs comparing with CBs, Kabir et al. [51] provide a comprehensive assessment of IBs’ liquidity risk and CR compared to CBs during the financial crisis. While Chamberlain et al. [23] investigate the differences in the credit profiles of IBs and CBs; this study extends this comparison to measure the link between CR and FP. Finally, while Riahi [89] investigates the impact of NPLs on the liquidity risk of both banks before and after the global crisis across GCC, we apply the same research but through moderating the pandemic of COVID-19.

The practical analysis supports and adds to the existing literature in different ways. First, to the best of our knowledge, this is the first study that linked NPL with FP and applies the comparison between IBs and CBs by moderating the impact of COVID-19. Second, this study uses a sample of MEA region banking industry before and during the recent crisis, while MEA region is mostly unexamined. Third, we contribute to the growing literature regarding the effects of COVID-19 on the financial markets (e.g., [27]). Our study adds to this strand of literature by showing that COVID-19 is an important driving factor of FP and CR. COVID-19 has elevated consciousness of significance of multiple factors that influence businesses’ FP [81]. Hence, we respond to this call. We supplementarily display that this widening consequence is felt most strongly in emerging economies as MEA region.

We were motivated by the work of Hunjra et al. [43] who investigated the link between FP and NPL and asking for the upcoming research by applying a comparison between CBs and IBs. This study is similarly inspired by Abdelaziz et al. [1] who claim that banks take diverse risks, which affect their FP differently. So far, papers on the consequence of risk and FP are focused outside developing economies. We motivated to apply the comparison between CBs and IBs as the IBs is one of the fastest growing sectors of the global financial industry [23].

Our purpose is twofold. First, we measure the impact of NPLs on FP. Second, we study for what extent this association differs in IBs comparing with CBs by providing comprehensive evidence on the impact of pandemic like COVID-19 as a mediator. To achieve these objectives, we used data of 200 banks across the MEA region for three years (2018–2020). Our sample was divided into two groups: 33 IBs and 167 CBs across 15 countries. The results find a negative association between NPLs and FP before and during the pandemic. Moreover, related to the variance between IBs and CBs, our analysis confirms the negative impact of CR over FP for the two groups. Our analysis reveals that COVID-19 is partially mediated the association between NPLs and FP across CBs while not across IBs. The rest of our research proceeds as follows: Section  2 sightsees the concept of CR in IBs relative to CBs. Section 3 demonstrates the theoretical framework behind the association between CR and FP. Section 4 reviews the literature and develops our hypotheses. Section 5 presents the sample selection, measurements of variables, models and adopted methodology. Section 6 discusses and analyses the results; then, the last section details the conclusions.

## Credit risk in IBs relative to CBs

There are robust grounds for believing that there are main variances between IBs and CBs. Zarrouk et al. [107] claim that interest rates are the rudimentary inspirations for circulation of money across traditional economies, while the demand for factual investments and use money for interchange capitals facilitate the progress of Islamic economies. Relative to CBs, IBs is more significant in economic progress as it incorporates supplementary characteristics like ethical standards, risk-sharing and operative CG. Turk and Sarieddine [102] highlight that IBs are subject to definite fiduciary, price and displaced marketable risks to which CBs counterparts are not. Risk-sharing in IBs among stakeholders is fairly diverse from that which happens in CBs. For Khan and Ahmad [57], Sharia executes distinctive characteristics on IBs, signifying that specific risk management is essential. Risk includes a number of forms and originates from a diversity of sources. One of these risks in IBs and CBs system is CR. Miscarriage to manage CR can damage image and FP and then the health of banks and the whole banking system.

In IBs, borrowers, depositors and the bank as intermediate share the incomes and risks of a loaning or investment transaction. There is an agreement between the bank and their depositors to use their money to funding the borrowers for the investment or loan. Depositors sign an unrestricted Mudaraba agreement, which permits the bank to use the credits and share the income of loans it makes to its debtors for investment (Musharaka). Portion of the risk ascending from investment’s possible failure hence moves to investor and bank. The profit to investors for Cihak and Hesse [25] must imitate the risk borne. Moreover, using of collateral to decrease CR is not obtainable to banks below the profit-sharing allowances. Most of the investments that made by IBs are not depend on profit-loss-sharing, however, rather, are debt-like in character [11].

Indication proposes that the interest-free nature of IBs obliges distinct services and products that can be concurrently considered as part of asset and liability characteristics. IBs seem to be unprotected toward the traditional banking risks, as their CBs. IBs for Boumediene [21] are uncovered to even additional risks as a result of their Sharia-compliant mechanisms implemented. Two risks are distinctive for IBs, particularly, the risk of return in addition to the displaced profitable risk, which ascends from their balance sheet incongruities. Hassan and Jemma [41] claim that capital adequacy ratio (CAR) serves as a crucial cushion in contradiction of IBs’ bankruptcy. Measuring IBs’ minimum capital requirements is applicable as a result of approved principle of profit and risk-sharing, which could decrease their whole risk. This principle supports significantly in promoting the depositor’s contribution in equity capital, therefore provocative diligence in the investment management and active control, which diminish the opposing selection and moral hazard hitches to minimalize the CR for IBs [97].

It does not certainly track that consequences for CR exposure are the identical for IBs as they are for CBs. The Islamic loans are designed inversely than the conventional one and are ruled through diverse agreements. Customers may have dissimilar motives for preferring one form of banking over the other, comprising religiosity, suitability or product category. Religiosity may have a behavior on default rates. Hilary and Hui [42] show a positive link between religiosity and aversion to risk. Debtors may be more probable to achieve their responsibilities under the Islamic loan agreements [11].

However, there is a more toughened clarification of IBs than that of distribution entirely profits and losses. Dridi and Hassan [29] claim that income is satisfactory, providing it is depending on a risk-sharing corporate. They go to differentiate substitute vehicles for circulation risk and distinguish among IBs according to the conducts in which risk is pooled in their business models. The preparations of bank have with its savers can similarly affect the sum of equity it has to increase, favoring to attain funds from deposits throughout periods of growing. Olson and Zoubi [82] argue that risk-sharing inspires depositors to manage the performance of IBs more carefully than they would CBs.

## Theoretical framework

NPLs are thought to degrade asset quality, raising risks in the bank's portfolios and lowering the return. NPLs are a standard indicator to assessing CR, and they have a direct impact on bank FP [61]. Excessive NPL portfolios weaken banks' capacity to profit, causing them to become weak and bankrupt. The paper was directed by three theories.

### Modern portfolio theory

Modern portfolio theory (MPT) is an investment theory that aims to explain how diversification in assets can help investors optimize their returns while minimizing their risks [70]. MPT is concerned with the creation of portfolios that optimize projected returns while remaining within an individual's risk tolerance. In bank performance studies, the portfolio theory method is the most relevant and plays a vital role. The banks should diversify their investment portfolios to reduce the CR takers defaulting on loan repayments and causing NPLs portfolios that affect profitability [73].

### Theory of asymmetric information

Information asymmetry is one of the pillars of Akerlof's lemon theory, which occurs when owners or managers have a better understanding of the risks and rewards associated with their business than lenders have [24]. In the financial market, asymmetric information is a concern; the distinguishing between good and bad borrowers might be difficult. Therefore, there may be issues with adverse selection and moral hazards. Pre-contractual information asymmetries cause adverse selection, but post-contractual knowledge asymmetries cause moral hazard [72]. When the profitability of a loan is determined by the type of borrower and interest rate, adverse selection emerges. This is due to the fact that higher interest rates tend to attract fewer acceptable borrowers. Such borrowers usually invest in high-risk ventures and have a higher chance of defaulting [64]. Lenders who provide credit to borrowers’ experience ambiguity about loan repayment because they cannot view the borrower's features and activities, making it difficult to determine the borrower's creditworthiness. Therefore, low-quality borrowers are supplanted by high-quality borrowers, resulting in a decline in the overall quality of bank loan portfolios, the accumulation of NPLs, decreased profitability [36].

The bad management hypothesis proposed by Berger and De Young [14] states that in response to an increase in NPLs, management tends to devote more resources to managing bad loans, resulting in an increase in operating expenses over interest income. As a result, a greater cost-to-income ratio indicates poor bank management in terms of loan underwriting, monitoring, and control [60]. The moral hazard dilemma states that a borrower is more likely to default if there are no consequences for future credit applications. This is due to lenders' difficulty estimating the amount of wealth borrowers will have accumulated by the due date of the debt, rather than at the time of application. If lenders are unable to determine a borrower's wealth, the latter may be inclined to default. To prevent this, lenders will raise interest rates, eventually leading to the market's collapse. Due to adverse selection and moral hazard, banks have accumulated a considerable amount of NPLs.

### Credit default theory

The credit default theory is applicable in instances where there is an indirect relationship between the effect of default on a bank's FP. This theory appears to be in line with studies on the relationship between NPL and FP, as it acknowledges “delinquency” and “insolvency” as the causes of NPL. Delinquency is described as failing to pay a debt on time, but insolvency is defined as having assets that are fewer than liabilities. The principle of delinquency is at the heart of the phrase credit default. This occurs when a borrower is unable to repay a loan on the due date due to a lack of liquidity. Delinquency initiates a solvency review, which may result in a negative equity position, resulting in loan cancelation and the lender's expectation of loss [85].

## Literature review and hypothesis development

### Impact of NPL on FP

Low CR in corporate activities is a predictor of successful governance procedures, which contribute to good financial outcomes [10]. The impact of NPLs on a bank's FP can be linked to a probable bank failure, a barrier to additional lending, and a drop in profit. Several studies have focused on this relationship. For example, Koju et al. [61], Ekinci and Poyraz [31] have examined the influence of NPL on banks' FP and have come up with mixed results. NPLs were cited as one of the major causes of the global financial crisis, which damage the USA economy and the economies of many other countries. Similarly, in COVID-19 pandemic, NPLs become a prominent topic and one of the important issues. NPLs can come from a variety factors; banks should be aware of them and take the appropriate actions to eliminate them from the industry.

The existing literature investigating the relationship between CR and FP can be divided into three groups: the first group found a negative relationship (e.g., [43, 75]). The second group finds a positive relationship (e.g., [30, 77]), while the third group suggests that no relationship (e.g., [34]). The results tend to differ depending on aspects as the context, metrics used or time period covered. Thus, the results could not easily be generalized, which gives a contribution for this study. Since interest income from bank assets forms an important component of a bank’s net income, impaired loans, or poor asset quality shows adversely on profitability. Most of the literature supports the negative relationship between NPL and FP. For example, Das and Uppal [28] found that NPL has negative influence on the rate of profit of the Indian banks. They advocate that the banks should decrease their NPLs as well as operating cost to recover their profitability. Similarly, Hunjra et al. [43] show that NPLs ratio has a negative impact on the FP of banks across South-Asia countries. Likewise, Munangi and Bongani [75] support the same impacts for South African banks. Ekinci and Poyraz [31] based on Turkey showed that there is a negative relationship between CR and FP. Musneh et al. [74] provide indication on the significance of risk in clarifying the cross-sectional stock returns difference in the industrial services and products sector on Bursa Malaysia. Several studies approved this negative association across different contexts (e.g., Abdelaziz et al. [1] for MENA region, Bishnu [18] for Nepal, Kingu et al. [60] for Tanzania, Isanzu [46] for China).

Athanasoglou et al. [9] explain this negative relation in the banking system; managers trying to maximize profits appear to have adopted a risk-averse strategy. According to Gropp et al. [39], excessive credit expansion, lending quality issues and poor CR management contributed to the global financial crisis. Leung et al. [66] mention that, during the financial crisis, banks with lower earnings have higher risks. Due to the insufficiency of previous accords, as Basel I and Basel II, Basel Committee on Banking Supervision adopted Basel III to deal with CR during the financial crisis [48]. Saif-Alyousfi [92] based on 45 CBs and 25 IBs indicate that Yemen War has a negative impact on deposits and loans of GCC banks. Pan et al. [86] across 78 countries show how CR has broadened significantly in response to COVID-19. One-percent increase in COVID-19 infections leads to 0.17% increase in CR spreads.

In contrast, Rajan [88] stated that credit policy is formed not only to make a profit but also to build a good reputation; as a result, bank aims to make credit policy from current earning to cover loan defaults in the future periods. Therefore, there is a positive relationship between NPLs and FP. Mushafiq et al. [77], based on Pakistan Stock Exchange, support this positive association. Duho et al. [30] found that CR is important in improving profit efficiency and ROE. Ozili [84] found that in the post-financial crisis period, there is a positive relationship between NPLs and FP. Nukala and Prasada Rao [78] support this association by applied covariance analysis to determine separate returns from two shares traded in S&P 500 and BSE Sensex indices. They indicate that returns not only augmented with growing beta values—but similarly increasing the growth rate of firm, earnings potential and stock price. The previous studies suggest that risk-averse shareholders target risk-adjusted returns and seek larger earnings to compensate higher CR.

However, Adebisi and Matthew [2] approve that there is no significant relationship between NPL and ROA. Similarly, Fang et al. [34] found that bank risk did not have a robust impact on FP metrics in China. Based on the previous works, there is still a gap in the research work. The previous work has been very conservative in explaining the relationship of CR and FP, and it has been limited to developed countries or one country or CBs rather than comparison with IBs and rather than considering the impact of the contemporary crisis. This gap is bridged by this study. Therefore, we formulate our H1.

**H1.** The association between NPLs and banks’ FP is negative before and during the COVID-19 pandemic.

### Islamic vs. conventional banks

The existing literature that compares between IBs and CBs for the FP and stability based on the CR can be divided into three clusters: the first cluster suggests that IBs are better than CBs in terms of FP and managing CR (e.g., [23], Safiullah [91]). The second cluster finds no difference between these banks (e.g., [8, 19]), while the third cluster suggest that CBs are better than those IBs (e.g., [58]).

Despite the significant differences between IBs and CBs, the literatures support the differences and similarities between the two clusters in terms of the impact of crisis or pandemic on the performance, behavior and other business decisions. In relation to the literatures that find differences between the two groups, Khasawneh [58] shows that IBs are more profitable than CBs, while CBs are more stable than IBs. Trad et al. [101] show that the effect of some banks’ characteristics is not the same for the CBs relative to IBs. For Alexakis et al. [8] based on GCC region, IBs have worse cost and profit performance than CBs, but they are on a par with regards in terms of performance. Jubilee et al. [50] based on 66 IBs and 319 CBs from 18 countries found that IBs are more productive than CBs, and the results from *t* test are further confirmed by the results from nonparametric tests. Kabir et al. [51] find that IBs outperform CBs in managing liquidity and CF. Safiullah [91] finds that IBs have 5.30% higher stability efficiency compared to CBs. Many studies support the variances between the two groups (e.g., Ferhi [35]). While other studies find consistencies between the two groups, Bourkhis and Mahmoud [22] conclude that there is no difference in the impact of crises on bank safety. Similarly, Bokhtiar et al. [19] find that COVID-19 creates identical volatility in both stock markets. Nomran and Haron [79] reveal that the returns of Islamic indices begun to be positive instead of negative by mid-April 2020, while returns of conventional ones remain negative. They suggest a negative significant impact of COVID-19 on the performance of both stock indices. Nevertheless, this impact is weak on the Islamic and strong on the conventional ones. The findings indicate that Islamic indices perform better before and during COVID-19 than the conventional ones. Therefore, we expect a different consequence for COVID-19 over the two groups when investigating the link between CR and FP.

**H2.** The association between NPLs and banks’ FP during the COVID-19 pandemic differs for IBs relative to CBs.

## Methods

### Sample

Our sample includes largest 200 banks (43 IB and 157 CB) according to the Asian Banker website (https://www.theasianbanker.com/ab500/2018-2019/largest-banks-mea) across 15 countries from the Middle East and the Africa (MEA) region from 2018 to 2021. Our sample includes Bahrain, Kuwait, Jordan, Qatar, Lebanon, Oman, Saudi Arabia and the United Arab Emirates in the Middle East and Egypt, Kenya, Ghana, Mauritius, Nigeria, Morocco, and South Africa in Africa. The sample does not appear to cover the full range of IBs and CBs across MEA region; we consider it to stand as a largest bank based on the size of assets. Our sampling of this period is solely based on the fact that it has never been considered in the previous studies to assess the NPLs in the banking sector during the COVID-19 pandemic. Consequently, we select one year before the pandemic and two years through the pandemic. Previous studies that evaluate NPLs considering data from current years and throughout the current pandemic are lacking. With a total of 800 observations, 12 out of the 15 countries incorporate IBs and CBs. The bank-specific data like size and capital adequacy have been collected from the banks’ annual reports and Asian Banker database. The macroeconomic variables are gathered from the Our World in Data.

### Methodology and model estimation

In line with the previous literature, we used panel ordinary least squares (OLS) with fixed and random effect, as well as the Hausman test to compare between the random effects model and the fixed effects models.

#### The fixed effect model (FEM)

Fixed effect model (FEM) assumes individual specific coefficient $\beta_{1i}$

1 $$\begin{aligned} & {\text{FP}}_{{it}} = \beta _{{1i}} + \beta 2{\text{CR}}_{{it}} + \beta 3{\text{COV}}_{{it}} + ~\beta 4{\text{FC}}_{{it}} + \beta 5{\text{MF}}_{{it}} + u_{{it}} \\ & i\, = \,1,2,3 \ldots 200\quad T\, = \,1,2,3,4 \\ \end{aligned}$$

where *i* in $\beta$ refers to the intercept values for each cross-section unit that may be different, ${\text{FP}}_{it}$ denotes the dependent variable (profit of the bank), ${\text{CR}}_{it}$ represents the explanatory variable-credit risk, ${\text{COV}}_{it}$ refers to the COVID-19 pandemic as a mediator, ${\text{FC}}_{it}$ represents control variables for firm characteristics, as size, ${\text{MF}}_{it}$ represents control variables for macroeconomic factors, as inflation, $\beta_{1i}$ is a constant term, while *β*2, *β*3, *β*4 and *β*5 represent coefficients, and $u_{it}$ refers to the error term.

#### Random effects model (REM)

REM model is assumed that the variation across entities is a random variable that is uncorrelated with the explanatory variables. As a result, the REM equation can be proceeded with Eq. (2):

2 $${\text{FP}}_{it} = \beta_{1i} + \beta 2{\text{CR}}_{it} + \beta 3{\text{COV}}_{it} + \beta 4{\text{FC}}_{it} + \beta 5{\text{MF}}_{it} + u_{it}$$

REM $\beta_{1i}$ is a random variable with a mean value of $\beta_{1}$ (no *i* subscript here), and the intercept of any cross-section unit is expressed as:

3 $$\beta_{1i} = \beta_{1} + \varepsilon_{i}$$

where $\varepsilon_{i}$ is a random error term with mean zero and variance $\sigma_{\varepsilon }^{2}$. Consequently, we can conclude that the 200 banks in our sample have a common mean value for the intercept equal $\left( {\beta_{1} } \right)$. The individual differences in the intercept values of each bank are reflected in the error term $\varepsilon_{i}$.

Substituting Eq. (3) into Eq. (2), we obtain

4 $$\begin{aligned} & {\text{FP}}_{it} = \beta_{1i} + \beta 2{\text{CR}}_{it} + \beta 3{\text{COV}}_{it} + \beta 4{\text{FC}}_{it} + \beta 5{\text{MF}}_{it} + u_{it} \\ & \quad \quad \quad \beta_{1i} + \beta 2{\text{CR}}_{it} + \beta 3{\text{COV}}_{it} + \beta 4{\text{FC}}_{it} + \beta 5{\text{MF}}_{it} + w_{it} \\ \end{aligned}$$

where

5 $$w_{it} = \varepsilon_{i} + u_{it}$$

The composite error term $w_{it}$ consists of two components: $\varepsilon_{i}$, which is the cross-section, or individual-specific, error component, and $u_{it}$, which is the combined time series and cross-section error component and is sometimes called the idiosyncratic term because it varies over cross-section (i.e., subject) as well as time. The error components model (ECM) is so named because the composite error term consists of two (or more) error components.

Since $\varepsilon_{i}$ is a component of $w_{it}$, it is possible that the latter is correlated with the explanatory variables. If that is indeed the case, the ECM will result in inconsistent estimation of the regression coefficients. So we use the Hausman test, which will tell us if $w_{it}$ is correlated with the explanatory variables, that is, whether ECM is the appropriate model or not [40].

#### Model estimation

To test our objectives, we have two clusters of hypotheses. The first used to measure the impact of CR on FP for the whole period, while the second one to investigate the impact of COVID-19 on the link between CR and FP. Each group includes three hypotheses: (1) the whole sample, (2) IBs and (3) CBs as follows:

**Model 1: impact of NPLs on FP for the whole sample and period**

6 $${\text{FP}}\left( {{\text{ROA}} - {\text{ROE}}} \right) = \beta 0 + \beta 1\;{\text{G}}.{\text{NPL}} + \beta 2\;{\text{SIZ}} + \beta 3\;{\text{LD}} + \beta 4\;{\text{EA}} + \beta 5\;{\text{CA}} + \beta 6\;{\text{LLR}} + \beta 7\;{\text{LA}} + \beta 8\;{\text{INF}} + \beta 9\;{\text{UNE}} + \varepsilon$$

**Model 2: impact of NPLs on FP for IBs and the whole period**

7 $${\text{FP}}\left( {{\text{ROA}} - {\text{ROE}}} \right) = \beta 0 + \beta 1\;{\text{G}}.{\text{NPL}} + \beta 2\;{\text{SIZ}} + \beta 3\;{\text{LD}} + \beta 4\;{\text{EA}} + \beta 5\;{\text{CA}} + \beta 6\;{\text{LLR}} + \beta 7\;{\text{LA}} + \beta 8\;{\text{INF}} + \beta 9\;{\text{UNE}} + \varepsilon$$

**Model 3: impact of NPLs on FP for CBs and the whole period**

8 $${\text{FP}}\left( {{\text{ROA}} - {\text{ROE}}} \right) = \beta 0 + \beta 1\;{\text{G}}.{\text{NPL}} + \beta 2\;{\text{SIZ}} + \beta 3\;{\text{LD}} + \beta 4\;{\text{EA}} + \beta 5\;{\text{CA}} + \beta 6\;{\text{LLR}} + \beta 7\;{\text{LA}} + \beta 8\;{\text{INF}} + \beta 9\;{\text{UNE}} + \varepsilon$$

**Model 4: impact of NPLs on FP for the whole sample after moderating **COVID**-19**

9 $${\text{FP}}\left( {{\text{ROA}} - {\text{ROE}}} \right) = \beta 0 + \beta 1\;{\text{G}}.{\text{NPL}} + \beta 2\;{\text{SIZ}} + \beta 3\;{\text{LD}} + \beta 4\;{\text{EA}} + \beta 5\;{\text{CA}} + \beta 6\;{\text{LLR}} + \beta 7\;{\text{LA}} + \beta 8\;{\text{INF}} + \beta 9\;{\text{UNE}} + \beta 10\;{\text{TC + }}\beta 11\;{\text{TD}} + \varepsilon$$

**Model 5: impact of NPLs on FP for IBs after moderating **COVID**-19**

10 $${\text{FP}}\left( {{\text{ROA}} - {\text{ROE}}} \right) = \beta 0 + \beta 1\;{\text{G}}.{\text{NPL}} + \beta 2\;{\text{SIZ}} + \beta 3\;{\text{LD}} + \beta 4\;{\text{EA}} + \beta 5\;{\text{CA}} + \beta 6\;{\text{LLR}} + \beta 7\;{\text{LA}} + \beta 8\;{\text{INF}} + \beta 9\;{\text{UNE}} + \beta 10\;{\text{TC + }}\beta 11\;{\text{TD}} + \varepsilon$$

**Model 6: impact of NPLs on FP for CBs after moderating **COVID**-19**

11 $${\text{FP}}\left( {{\text{ROA}} - {\text{ROE}}} \right) = \beta 0 + \beta 1\;{\text{G}}.{\text{NPL}} + \beta 2\;{\text{SIZ}} + \beta 3\;{\text{LD}} + \beta 4\;{\text{EA}} + \beta 6\;{\text{CA}} + \beta 7\;{\text{LLR}} + \beta 8\;{\text{LA}} + \beta 9\;{\text{INF}} + \beta 10\;{\text{UNE}} + \beta 11\;{\text{TC + }}\beta 12\;{\text{TD}} + \varepsilon$$

Under each model, we measured FP using ROA (return on assets) and ROE (return on equity). Also, we measured the impact of COVID-19 by total number of cases (TC) and by total number of deaths (TD). While G.NPL refers to gross NPL ratio %, SIZ refers to the firm size based on log of total assets, LD refers to loan to deposit ratio%, EA refers to equity to assets ratio %, CA refers to capital adequacy ratio total, LLR refers to loan loss reserves to gross NPLs %, LA refers to liquid assets to total deposits and borrowings%, INF refers to inflation% CPI, UNE refers to the unemployment, total (% of total labor force).

### Definitions and measurement of variables

The independent variable is NPLs ratio and adopted as an indicator of CR. It reflects the probability of the borrowers of a bank not being capable to meet the financial debts [30]. The upper NPL, the inferior of credit quality and, consequently, the greater risk that more loan loss will be charged contrary to income. Here, the NPLs were measured as a proportion of NPLs to total loans. Several studies use this ratio to measure CR (e.g., [43, 64]).

The dependent variable is FP. The accounting metrics that adopted to measure FP are return on asset (ROA) and return on equity (ROE). ROA is used for measuring the profitability of the banks and calculate as the net income over the total assets. While ROE is calculated as which is net income over the shareholders’ equity. High ROA displays that the bank’ financial position is steady, and they are not absorbed in investing in risky loans as of less pressure for make income. Adopting these ratios can be verified in the literature (e.g., [16], Tan et al. 2017). Similarly, several authors apply these accounting measurements throughout the COVID-19 (e.g., [96, 106]). The moderating variable is COVID-19 pandemic. To measure the greatness of this pandemic, we use the annual confirmed cases and annual deaths by country which collected from the Our World in Data. Our World in Data gathers coronavirus associated data from Johns Hopkins University Coronavirus Resource Center, and it adopted before by Pan et al. [86].

The literature has recognized that there is a countless of factors (bank-specific, industry-specific and country-specific), which could affect the banks’ performance and the link between CR and FP (e.g., [12, 16, 83]). The controlled variables for the FP include firm size, loan-to-deposit ratio%, cost-to-income ratio%, net interest income to total assets %, equity-to-assets ratio %, capital adequacy ratio %, capital adequacy ratio total, loan loss reserves to gross NPLs %, liquid assets to total deposits and borrowings% as a country-level variables, while inflation and unemployment are used as a country-level variables.

The positive influence of bank size on FP has been informed by Muhammad Mushafiq et al. [77]. Large businesses have more competitive power relative to small companies according to superior access to capital, larger market share, and operational efficiencies [45]. For Levy [67], COVID-19 has augmented the incomes for huge pharmaceutical and technological corporations, whereas aching or bankrupting countless smaller firms. Mushafiq et al. [77] found that leverage is greatly significant and validates an opposite association with FP, which is similar for Akinlo and Asaolu [6]. This may be explained as the businesses’ obligation increases the drop in FP, which indicates the riskier condition will result in poorer FP. The corporation is more susceptible to insolvency as financial borrowing upsurges in circumstances which is not very decent. For Gadzo et al. [36], the efficiency by using cost-to-net income ratio has significantly positively effect on the FP. The more effective bank is the more incomes it creates.

Kwashie and Awadzie [63] found that net interest income-to-bank assets ratio displays a positive association with FP that should attempt to progress on loan performance to upsurge the profitability. Similarly, a greater quality of loans would result in growth in net interest income and for that issue upper FP. To improve the FP, banks should decrease the spread of interest rate. This is because a great interest rate banquet has the propensity to influence negatively on FP of the bank. When the loan loss provision decreases the operating expenditures to operating income upsurges. Consequently, loan loss to go down more expenditure has to be made in relative to profit [63].

Kingu et al. [60] found a negative link between liquidity based on loan to deposit ratio and liquid assets and FP. It indicates that bank is revealing itself to financial distress and liquidity risk when the liquidity rises. A higher proportion gives the impression that the bank has reached its edge of funding loans from its own credits, and uses costlier techniques as costly deposits, equity and debt financing to fund its loan that decreases its FP. CAR reflects the protection depositor’s contrary to unsuspected losses. Literature has revealed that CAR can be positively or negatively or interrelated to FP. For instance, Peter et al. (2018) show a positive association, whereas et al. (2016) reported negative link, which specifies that greatly capitalized banks make less FP. Ezike and Oke [32] identified that keeping capital beyond the optimum level would contrariwise affect the FP. The outcomes show an upsurge in capital adequacy ratio that has a power over the rising movement of the profits.

As our sample is cross countries, we include two macroeconomic factors: inflation and unemployment. The performance of corporate could not be unglued from the inspiration of the macroeconomic influences. Inflation refers to the level at which broad price increases in the economy during a year. Precise forecast of inflation can have a positive influence on FP (e.g., [85]). These literatures show that greater inflation declines the value of outstanding debts, which progresses the repayment capacity of the businesses. Unemployment refers to the state in the economy in where assets are not involved in the country’s productive activities or when they are under-involved. John [49] supports a negative link between FP and unemployment (Table 1).

**Table 1:** Variables and definitions

Variables	Proxies	Definition/calculation	Source
**Dependent variable**			
Financial performance (FP)	Return on assets (ROA)	Operating profit/average total assets	Annual reports and Asian Banker website
	Return on equity (ROE)	Net profit/average total equity	
**Independent variable**			
Credit Risk (CR) or G.NPL	Gross nonperforming loans (NPLs) ratio	Nonperforming loans/total loans	Annual reports and Asian Banker website
**Moderating variable**			
Pandemic	COVID-19 pandemic	Number of total cases by country Number of total deaths by country	Our World in Data
**Control variables for microeconomic level**			
LD	Bank liquidity	Loan to deposit %	Annual reports and Asian Banker website
EA	Financial leverage	Equity to assets %	
CA	Stability	Capital adequacy ratio total	
LLR	Quality of loans	Loan loss reserves to gross NPLs %	
LA	Liquidity	Liquid assets to total deposits and borrowings %	
SIZ	Size of bank	Natural logarithm of total assets	
**Control variables for macroeconomic level**			
UNE	Unemployment	Unemployment, total (% of total labor force) (modeled ILO estimate)	World bank database
INF	Inflation	Annual rate of consumer price index inflation	

## Results and discussion

### Descriptive statistics

Table 2 represents that the mean values for NPL, ROA and ROE were 7.26, 1.398, and 11.126, respectively. The mean value of FP indicates that the banks are acting well and are making incomes. We applied a paired t-test to explore to what extent there is a significant difference between IBs and CBs for NPLs. Our findings reveal that there is a significant variance in NPLs for both groups. The average NPL ratio for the whole period as presented in Table 3 is equal to 4.29 for IBs and 7.84 for CBs. Therefore, IBs have lower NPLs across the whole period, before and after the COVID-19 period; the variance is statistically significant at 1%.

**Table 2:** Descriptive statistics

	Minimum	Maximum	Mean	SD
Gross NPL%	0.150	65.66	7.69	8.49
Return on equity %	− 89.60	44.200	10.58	10.74
Return on assets %	− 9.500	8.300	1.381	1.499
Total COVID-19 cases	0	3,458,286	219,988	504,713
Total COVID-19 deaths	0	91,145	4152.9	12,836
Inflation% CPI	− 2.5403	84.864	4.230	9.274
Unemployment	.110	28.74	7.898	6.754
Firm size	4.56	12.548	8.827	1.555
Loan-to-deposit ratio%	10.50	32,416.70	121.22	1234.3
Equity-to-assets ratio %	2.10	69.2	13.110	5.125
Capital adequacy % total	3.700	166.8	19.36	8.249
Loan loss reserves to gross NPLs %	2.70	747.3	105.69	79.207
Liquid assets to total deposits and borrowings %	5.70	231.9	38.12	20.353

*ROA* return on equity %; *ROE* return on assets %; *G.NPL* gross NPL ratio %,; *SIZ* firm size based on log of total assets; *LD* loan-to-deposit ratio%; *EA* equity-to-assets ratio %; *CA* capital adequacy ratio total; *LLR* loan loss reserves to gross NPLs %; *LA* liquid assets to total deposits and borrowings%; *INF* inflation % CPI; *UNE* unemployment, total (% of total labor force) (modeled ILO estimate); *TC* total cases of COVID and *TD* total deaths of COVID

**Table 3:** NPLs, ROA, and ROE before and during COVID-19 pandemic for IBs and CBs

	Islamic Banks			Conventional Banks		
	Whole period	Prior COVID-19	During COVID-19	Whole Period	Prior COVID-19	During COVID-19
Gross NPL%	4.29	3.59	4.74	7.84	3.64	5.58
ROE	8.988	10.003	8.511	7.870	12.839	7.809
ROA	0.755	1.200	0.545	0.935	1.565	0.900

*ROA* return on equity %; *ROE* return on assets % and *G.NPL* gross NPL ratio %

During the pandemic era, the NPL increases for CBs (5.85) more than IBs (4.74). This result is matching with Chamberlain et al. [23] who find that IBs have lower CR relative to CBs. It is also supporting the work of Khediri et al. [59] who indicated that IBs have lower CR during and after the crisis. It justifies as IBs’ system circumvents the high-risk investments as it is more of profit management and conservative. Therefore, CBs tends to have a slighter risk than IBs. This is an indication that the weakness in risk management practices among some CBs comparing with IBs led to greater NPLs. Our result is in contradiction of Grassa [38] who shows that Islamic profit-loss sharing (PLS) products present superior liquidation risk comparing with CBs, and this type of risk has a more detrimental impact on performance during a crisis.

We conduct similarly paired t-test to explore the variance between IBs and CBs toward FP. Our findings reveal that there is a significant variance for the two groups. IBs do not have higher average FP as measured by ROE than CBs do. For IBs, the ratio has a mean of 8.988%; for CBs, the ratio has a mean of 7.870%. During the COVID-19 period, the average ROE declined from 10.003 to 8.511% for IBs and the average ROE for CBs similarly deteriorated from 12.839 into 7.809%. This result is matching with Khasawneh [58] who find CBs have FP higher than IBs during the crisis. We can conclude that COVID-19 has a negative influence on FP for both IBs and CBs. This result is supported by Bourkhis and Mahmoud [22] who find that CBs and IBs were affected thru the global financial crises in terms of FP and stability.

The mean for ROA shows that FP of CBs during the pandemic is higher than IBs; these findings are inconsistent with Khediri et al. [59]. Possible justifications may refer to the different way of working, where IBs are subject to Sharia, as it does not deal with interest, but focus on real investment. Therefore, in case of pandemic like COVID-19, the amount of investment reduced and fearing behavior for investor is becoming the priority, which have the effect directly on the performance of the banks. This has been confirmed by Wasiuzzaman and Gunasegavan [104]. Related to mean of COVID-19 cases and deaths for our selected countries, Table 2 shows that the mean values for total cases and total deaths were 68,585.39 and 1258.40, respectively. These numbers are matching with the global average cases and deaths rates. Also, unit root tests, such as the Levin, Lin&Chu t-stat, Fisher ADF test, and Fisher PP test, were used to examine the relationship between the variables. Table 4 shows that at that level, all variables are stable.

**Table 4:** Panel unit root tests results in level

	LLC	ADF-fisher	PP-fisher
ROA	− 168.46***	582.03***	698.76***
ROE	− 19.84***	575.34***	686.39***
LA	− 2983.9***	638.11***	756.28***
G.NPL	− 10,645.4***	475.31***	555.78***
LLR	− 178.91***	480.67***	576.54***
CR	− 36.72***	480.70***	573.68***
EA	− 81.57***	590.75***	714.84***
LD	− 85.01***	575.48***	664.61***
SIZ	− 180.86***	806.51***	902.96***
UNE	− 1282.7***	667.39***	708.77***
INF	− 1653.05***	592.04***	667.37***
TD	− 334.86***	439.52***	496.32***
TC	− 2361.89***	423.91**	525.51***

^***^, **Mean significant at 1%, 5%

### Correlation matrix

Table 5 presents the information on the dependent as well as explanatory variables and their association to each other. We find that there is a negative association between NPLs and ROA as well as ROE. There is a negative relationship with size, loan loss reserves to gross NPLs and unemployment, whereas we found a positive association with cost to income, capital adequacy, equity to assets and liquid assets to total deposits. Furthermore, most of the association values are comparatively minor (less than 0.70), which proposes that there is no significant concern of multicollinearity. The multicollinearity problem exists when the association between the variables is higher than 0.9.

**Table 5:** Correlation analysis

	G.NPL	SIZ	LD	EA	CA	LLR	LA	INF	UNE	ROE	ROA	TC	TD
G.NPL	1	− .387^**^	− .002	.233^**^	.077^*^	− .312^**^	.235^**^	.099^*^	− .103^*^	− .195^*^	− .116^*^	.037	.012
SIZ		1	− .020	− .249^**^	− .197^*^	.271^**^	− .136^**^	− .062^*^	− .024	− .009	− .084	.076^*^	.025
LD			1	.170^**^	.680^**^	− .018	0.00^**^	− .036	− .022	− .020	.020	− .007	− .010
EA				1	.525^**^	− .086	.271^**^	− 0.08^*^	− .123^*^	.009	.283	− .000	− .015
CA					1	.010	− .219^**^	− 0.06	− .095	.191^**^	.350^*^	− .023	− .006
LLR						1	− .056^*^	− .034	− .061^**^	.044^*^	− .032	− .027	− .012
LA							1	.265^**^	− 0.005	.193^**^	.232^**^	− .038	.013
INF								1	.043	.102^**^	.080^*^	− .020	− 001
UNE									1	.012	− .034	.515^**^	.538^**^
ROE										1	.89^**^	− .180^**^	− .062
ROA											1	− .177^**^	− .080
TC												1	.936^**^
TD													1

^**^Correlation is significant at the 0.01 lev

^*^Correlation is significant at the 0.05 lev

*ROA* return on equity %; *ROE* return on assets %; *G.NPL* gross NPL ratio %; *SIZ* firm size based on log of total assets; *LD* loan-to-deposit ratio%; *EA* equity-to-assets ratio %; *CA* capital adequacy ratio total; *LLR* loan loss reserves to gross NPLs %; *LA* liquid assets to total deposits and borrowings%; *INF* inflation % CPI; *UNE* unemployment, total (% of total labor force) (modeled ILO estimate); *TC* total cases of COVID and *TD* total deaths of COVID

### Regression analysis

Table 6 shows the result of predictable relationship between NPLs and FP for the whole sample and the whole period (before and during the pandemic) according to model 1. Banks with a great level of income are less involved in hazardous investments, which may lead to NPLs in the future; therefore, we argue that there is a negative link between FP and NPLs. This proposes that an upsurge in CR decreases the value of ROE and ROA. This is clarifying why banks with high-risk-taking conduct have a great level of NPLs, leading to a negative impression on the FP. NPLs negativity affect ROA at 1% significant level and affects ROE at 10% significant level. This result proposes that to provide incomes to stockholders, there is a need to prudently manage CR through guaranteeing that bad loans are reduced. Consequently, we confirm H1. This specifies that the great provisioning of the NPLs could be lessening the banks’ FP. Munangi and Bongani [75], Hunjra et al. [43] reported the same results.

**Table 6:** Impact of NPLs on FP for the whole sample and period

Model	Model 1: Impact of NPL on FP for the whole sample and period					
	Impact of NPL on ROA for the whole sample and period Fixed effect			Impact of NPL on ROA for the whole sample and period Random effect		
	Coefficients	T	Sig	Coefficients	T	Sig
(Constant)	− 0.09	− 0.169	0.866	− 0.348	− 0.787	0.431
G.NPL%	− 0.043	− 5.041	0.000***	− 0.041	− 5.97	0.000***
SIZ	− 0.00004	− 0.007	0.994	− 0.008	− 0.222	0.823
LD %	− 0.0004	− 5.22	0.000***	− 0.0004	− 7.421	0.000***
EA %	0.021	1.49	0.136	0.019	1.630	0.103**
CA %	0.076	6.050	0.000***	0.094	9.059	0.000***
LLR %	− 0.002	− 2.360	0.018**	− 0.002	− 3.041	0.002**
LA %	0.008	2.24	0.025**	0.008	2.866	0.004**
INF	0.009	1.50	0.135	0.013	2.45	0.014**
UNE	− 0.006	− 0.56	0.57	− 0.004	− 0.564	0.573
Model summary	Adjusted R Square	0.529		Adjusted R Square	0.212	
	F	4.706		F	21.48	
	Sig	0.000		Sig	0.000	
Hausman test	Chiq 8.339	Prob	0.5004			

Model	Model 1: Impact of NPL on FP for the whole sample and period					
	Impact of NPL on ROE for the whole sample and period Fixed effect			Impact of NPL on ROE for the whole sample and period Random effect		
	Coefficients	T	Sig	Coefficients	T	Sig
(Constant)	7.93	1.873	0.062*	5.97	1.77	0.077*
G.NPL%	− 0.279	− 4.231	.000***	− 0.292	− 5.586	.000***
SIZ	− 0.297	− 0.765	0.444	− 0.237	− 0.808	0.419
LD %	− 0.002	− 4.28	.000***	− 0.0025	− 5.848	0.000***
EA %	− 0.313	− 2.84	.000***	− 0.345	− 3.750	0.000***
CA %	0.481	4.998	.000***	0.567	7.187	0.000***
LLR %	− 0.006	− 1.039	0.298	− 0.006	− 1.247	0.213
LA %	0.076	2.73	.006**	0.084	3.88	0.000***
INF	0.033	0.722	0.470	0.067	1.648	0.099*
UNE	0.016	0.212	0.832	0.0079	0.130	0.896
Model summary	Adjusted R Square	0.529		Adjusted R Square	0.129	
	F	4.706		F	12.386	
	Sig	0.000		Sig	0.000	
	Chiq 5.394	Prob	0.798			

This result has supported the argument of modern portfolio theory, which claims the banks should consider the varying investments portfolio to diminish CR takers nonpayment in loans repayments and causing NPL portfolios, which have the effect on FP. It is similarly reliable with the credit default theory, which states that when a debtor is incapable to refund a loan, rise of NPLs and creditor's anticipation of loss, may consequence in a negative equity site. Conflicting with the traditional finance theory that argues that the greater risk, the greater return, upper CR in the form of NPLs rather leads to inferior income. Thus, losses from NPLs rather corrode the income of banks leading to a bargain the whole bank income. Moreover, the negative influence of NPLs over FP displays to what extent managers in the banks across MEA region implements a risk-averse method commonly and during the crisis to maximize their profits. In the long run banks will own less assets to be adopted for generate interest income and will lead to their incapability to develop FP. Investigating this from the information asymmetry occupants of the lemon theory assumes that at any time there is information asymmetry between the customers seeking the loan and banks; it is expected to result into a high rate of NPLs.

The results of control variables related to model 1 indicate that NII to total assets, capital adequacy, liquid assets to total deposits and inflation positively effects on FP based on ROA and ROE. In the other side, the analysis shows negative influences of size, loan to deposit; cost to income, equity to assets and capital adequacy Tire 1 on banks’ FP. These results are matching with Kwashie and Awadzie [63], Ozogbuda [85] who show to what extent factors as inflation, liquidity and stability can improve the FP, while Mushafiq et al. [77], Esther et al. (2016) indicate that other factors like size, and leverage may harm the FP.

Table 7 presents the results of models 2 and 3, which splitting the sample into IBs and CBs. The analysis of model 2 shows the comparable results as in the first model by supporting a negative association with ROA at 1% and 10% level significant for ROE related to CBs. The analysis of model 3 displays the same analogous results by showing a negative association with ROA and ROE at 5% level significant for IBs. This result reflects to what extent the association between NPLs and FP does not change between IBs and CBs. This result is matching with Bourkhis and Mahmoud [22]. In the other side, this result rejects the debating of Trad et al. [101] who support a variance between IBs and CBs. This result shows that NPLs ratio has an exceptionally significant statistical meaning and a promising association to ROE and ROA, which shows that flagging business health stability in terms of CR contributes to damaging the FP. Consequently, we reject H2 according to the similarity of the result for the two clusters of banks.

**Table 7:** Impact of NPLs on FP for the conventional banks for the whole period

Model	Model 2: Impact of NPL on FP for CBs for whole period						Model 3: Impact of NPL on FP for CBs for whole period					
	Impact of NPL on ROA for the CBs Fixed effects			Impact of NPL on ROA for CBs Random effects			Impact of NPL on ROE for the CBs Fixed effects			Impact of NPL on ROE for CBs Random effects		
	Coefficients	T	Sig	Coefficients	T	Sig	Coefficients	T	Sig	Coefficients	T	Sig
(Constant)	** − **1.132	** − **1.86	0.063*	** − **0.551	** − **1.182	0.237	1.7431	0.381	0.703	4.154	1.151	0.250
G.NPL%	** − **0.024	** − **2.78	0.006**	** − **0.033	** − **4.87	.000***	** − **0.187	** − **2.936	0.003**	** − **0.255	** − **4.830	0.000***
SIZ	0.103	1.97	0.049**	0.008	0.202	0.839	0.384	0.979	0.327	** − **0.036	** − **0.116	0.908
LD %	** − **0.0003	** − **4.289	0.000***	** − **0.0004	** − **6.80	.000***	** − **0.002	** − **3.537	0.000***	** − **0.002	** − **5.292	0.000***
EA %	0.037	2.4435	0.015**	0.025	1.977	.048**	** − **0.232	** − **2.029	0.043**	** − **0.321	** − **3.283	0.001**
CA %	0.075	5.478	0.000***	0.093	8.527	.000***	0.458	4.462	0.000***	0.559	6.642	0.000***
LLR %	** − **0.003	** − **3.169	0.002**	** − **0.003	** − **3.38	.001**	** − **0.019	** − **2.583	0.010**	** − **0.013	** − **2.126	0.034**
LA %	0.0014	0.371	0.710	0.007	2.220	.027**	0.047	1.589	0.113	0.079	3.419	0.001**
INF	0.0109	1.531	0.126	0.013	2.209	.027**	0.029	0.548	0.584	0.057	1.291	0.197
UNE	0.0177	1.491	0.136	0.004	0.530	0.596	0.1707	1.915	0.056*	0.068	1.024	0.306
Model summary	Adjusted R Square	0.494		Adjusted R Square	0.228F		Adjusted R Square	0.444		Adjusted R Square	0.124	
	F	4.251		F	19.95		F	3.662		F	20.122	
	Sig	0.000		Sig	0.000		Sig	0.000		Sig	0.000	
Hausman test	Chi sq. 38.919	Prob	0.000				Chi sq. 26.793	Prob	0.001			

Model	Model 5: Impact of NPL on FP for IBs for whole period						Model 6: Impact of NPL on FP for IBs for whole period					
	Impact of NPL on ROA for the IBs Fixed effects			Impact of NPL on ROA for IBs Random effects			Impact of NPL on ROE for the IBs Fixed effects			Impact of NPL on ROE for IBs Random effects		
	Coefficients	T	Sig	Coefficient s	T	Sig	Coefficient s	T	Sig	Coefficients	T	Sig
(Constant)	− 0.485	− 0.334	.739	0.686	0.657	.512	1.789	0.168	.867	15.845	2.057	.042**
G.NPL%	− .151	− 5.35	.000***	− .169	− 7.719	.000***	− .599	− 2.901	.005**	− .812	− 5.026	.000***
SIZ	0.363	3.336	.001**	0.292	3.668	.000***	2.806	3.522	.000***	1.987	3.382	.001**
LD %	− .005	− 0.798	.428	− .009	− 2.198	.030**	− .119	− 2.757	.008**	− .130	− 4.111	.000***
EA %	− 0.063	− 1.169	.247	− .026	− 0.862	.390	− 0.683	− 1.714	.092*	− .379	− 1.722	.088*
CA %	.059	1.553	.126	0.029	1.166	.246	.455	1.614	.111	.012	0.065	.948
LLR %	− 0.001	− 0.91	.363	− 0.002	− 2.332	.0217**	0.013	1.474	.146	− 0.007	− 0.110	.912
LA %	− 0.033	− 2.65	0.010**	− 0.027	− 2.986	.004**	− .325	− 3.571	.000***	− .239	− 3.606	.000***
INF	.086	2.83	.006**	.071	2.922	.004**	1.123	5.065	.000***	1.029	5.717	.000***
UNE	− 0.039	− 1.484	.143	− 0.026	− 1.551	.124	− 0.058	− 0.301	.764	− 0.104	− 0.834	.406
Model summary	Adjusted R Square	0.778		Adjusted R Square	0.668		Adjusted R Square	0.684		Adjusted R Square	0.618	
	F	8.699		F	25.17		F	13.253		F	20.39	
	Sig	0.000		Sig	0.000		Sig	0.000		Sig	0.000	
	Chi sq. 9.359	Prob	0.405				Chi sq. 11.854	Prob	0.222			

Instead of uniqueness of IBs relative to CBs, the association between CR and FP remains the same. The insignificant variances can come back to the lack of commitment of IBs on applying PLS, which reduce the gap with CBs. Aggarwal and Yousef [4] argue that most of loans that provided by IBs are debt-like in character. For Rosly and Bakar [90] riba (Interest) is masquerading as credit financing, a finding echoed in the outcomes of Khan [55]. Baele et al. [11] find that less than 3% of loans, which delivered by IBs, were based on PLS principles. Furthermore, IBs like CBs are exposed to CR and effects negatively on their FP.

### Impact of COVID as a mediator over the link between NPLs and FP

We adopt Baron and Kenny's [13] regression method to examine whether COVID-19 pandemic mediates the association between NPLs and FP. Testing for mediation consequence can be achieved through three steps: (1) regressing the mediator toward the independent variables, (2) regressing the dependent variable toward the independent variables, and (3) regressing the dependent variable toward the two independent variables and mediator. Three substitutes were suggested through Baron and Kenny. First, if the influence of independent variables on the dependent variable becomes insignificant in the attendance of the mediator, then the independent variables' effects are totally mediated through mediator. Second, if the impact of independent variables is significant in the existence of mediator, the significances of the independent variable are partially mediated. There is no mediation consequence if the wholly beyond situations are not met.

Our findings for model 4 reveal that NPLs during COVID-19 are not considered as a primary and only determinant of firm's FP which is support our hypothesis that COVID-19 is treated as entirely mediated the relationship between NPLs and FP for the whole sample. As presented in Table 7, NPLs negativity affects ROA at 5% and affects ROE at 1% significant level during COVID-19. Consequently, both groups are negatively affected by the pandemic. This result shows to what extent the negative impact for NPLs over FP remains identical before and during the pandemic. This result is matching with Saleh and Abu-Afifa [93].

Table 8 shows the result of models 5 and 6. The analysis finds a negative association between NPLs and FP for IBs and CBs. This indicates that the profitability of banks is strongly influenced by increasing the amount of NPLs. Unlike our results, it shows that COVID-19 is partially mediated the relationship between NPLs and FP for CBs according to model 5, while COVID-19 is not treated as a mediator in case of IBs based on model 6. The different influence’ result for COVID-19 over CBs relative to IBs is not matching with Bokhtiar et al. [19] who find that COVID-19 creates similar volatility for IBs and CBs. Our result may has explained by the findings of Bilgin et al. [17] who find that growth in economic indecision like pandemic significantly declines the credit growth of CBs but does not have a significant influence over IBs’ credit growth as IBs are immune to financial vagueness.

**Table 8:** Impact of NPLs over FP after moderating COVID-19 for the whole sample

Model	Model 4					
	Impact of NPL on ROA by moderating the COVID-19 Fixed effect			Impact of NPL on ROA by moderating the COVID-19 Random effect		
	Coefficients	T	Sig	Coefficients	T	Sig
(Constant)	− 0.707	− 1.33	.183	− 0.919	− 2.150	.031**
G.NPL%	− .030	− 3.70	.000***	− .030	− 4.538	.000***
SIZ	.058	0.120	.229	0.053	1.429	.153
LD %	− .0004	− 5.546	.000***	− .0004	− 7.779	.000***
EA %	.027	1.980	.048**	0.027	2.365	.018**
CA %	.077	6.44	.000***	0.093	9.47	.000***
LLR %	− 0.002	− 2.45	.015**	− .002	− 3.314	0.001**
LA %	.005	1.33	.183	.005	1.75	.079*
INF	.0099	1.735	.083*	0.013	2.537	.011**
UNE	.021	1.898	.058*	.017	1.93	.053*
TC	− 1.82E − 06	− 6.87	.000***	− 1.92E − 06	− 7.84	.000***
TD	5.42E − 05	5.074	.000***	5.76E − 05	5.87	.000***
Model summary	Adjusted R Square	0.578		Adjusted R Square	0.283	
	F	5.476		F	25.608	
	Sig	0.000		Sig	0.000	
Hausman test	Chi sq. 11.299	Prob	0.418			

Model	Model 4					
	Impact of NPL on ROE by moderating the COVID-19 Fixed effects			Impact of NPL on ROE by moderating the COVID-1Random effects 9		
	Coefficients	T	Sig	Coefficients	T	Sig
(Constant)	3.098	0.762	.446	1.424	0.44	.659
G.NPL%	− .182	− 2.85	.004**	− .0210	− 4.181	.000***
SIZ	.136	0.364	.716	0.247	0.881	.378
LD %	− .0023	− 4.592	.000***	− .0025	− 6.217	.000***
EA %	− 0.272	− 2.603	.009**	− 0.287	− 3.292	.001**
CA %	.491	5.37	.000***	0.568	7.633	.000***
LLR %	− 0.006	− 1.00	.316	− .007	− 1.448	0.148
LA %	.050	1.87	0.06*	.058	2.83	.005**
INF	.039	0.897	.369	0.066	1.728	.084*
UNE	.245	2.878	.004**	.177	2.643	.008**
TC	− 1.30E − 05	− 6.38	.000***	− 1.49E − 05	− 8.004	.000***
TD	0.0003	4.35	.000***	0.0004	5.956	.000***
Model summary	Adjusted R Square	0.516		Adjusted R Square	0.215	
	F	4.486		F	18.123	
	Sig	0.000		Sig	0.000	
	Chi sq. 15.685	Prob	0.153			

Furthermore, the business models of IBs have topographies that can improve stability specially during the crisis era. Similarly, this result may justify based on the work of Samaoui et al. [94] who argue that banks with less funding liquidness risk seeks to involve in more risk-taking conduct, which cumulative their insolvency risk. IBs tend to be more careful about taking investing more risk than CBs. For Louati et al. [68], the capitalized CBs turn out to be more involved in an extreme risk-taking performance, causing in augmented toxic-loan ratios and, instantaneously, a rather shaken stability relative to IBs. The agency glitches within IBs might well stand at the basis of such a relationship. On the one hand, non-warranty obligation is used in confident Islamic financing transactions, as Musharaka may exacerbate the agency difficulties. On the other hand, IBs obliging to tolerate some intrinsic costs, created by failure of convinced financial projects in a participating process, are comparatively risky with the presence of applied problems these banks are expected to face in controlling and managing several projects (Table 9).

**Table 9:** Impact of NPLs on FP for convectional banks as well as Islamic banks after moderating the COVID-19

Model	Model 5						Model 6					
	Impact of NPL on ROA by moderating the COVID-19 for CBs Fixed effect			Impact of NPL on ROA by moderating the COVID-19 for CBs Random effect			Impact of NPL on ROE by moderating the COVID-19 for IBs Fixed effect			Impact of NPL on ROE by moderating the COVID-19 for IBs Random effect		
	Coefficients	T	Sig	Coefficients	T	Sig	Coefficients	T	Sig	Coefficients	T	Sig
Constant	** − **1.767	** − **3.070	0.002**	** − **1.173	** − **2.619	0.009**	** − **2.759	** − **0.633	0.526	** − **0.271	** − **0.078	0.937
G.NPL %	** − **0.0101	** − **1.239	0.215	** − **0.021	** − **3.256	0.001**	** − **0.089	** − **1.458	0.145	** − **0.168	** − **3.310	0.001**
SIZ	0.155	3.153	0.002**	0.072	1.880	0.061*	0.763	2.047	0.041**	0.422	1.432	0.152
LD %	** − **0.0003	** − **4.689	0.000***	** − **0.0004	** − **7.134	0.000***	** − **0.002	** − **3.831	0.000***	** − **0.002	** − **5.572	0.000***
EA %	0.0419	2.916	0.004**	0.034	2.8615	0.004**	** − **0.196	** − **1.809	0.071*	** − **0.251	** − **2.715	0.007**
CA %	0.0779	6.018	0.000***	0.093	8.934	0.000***	0.477	4.870	0.000***	0.558	6.988	0.000***
LLR %	** − **0.002	** − **2.555	0.011**	** − **0.0025	** − **3.409	0.001**	** − **0.013	** − **1.965	0.050**	** − **0.011	** − **2.045	0.041**
LA %	** − **0.002	** − **0.570	0.569	0.0030	1.060	0.289	0.021	0.754	0.451	0.052	2.388	0.017**
INF	0.0118	1.762	0.079*	0.0128	2.3657	0.018**	0.035	0.697	0.485	0.058	1.409	0.159
UNE	0.043	3.556	0.000***	0.027	2.9036	0.004**	0.349	3.782	0.000***	0.220	3.066	0.002**
TC	** − **1.92E − 06	** − **6.32	0.000***	** − **2.08E − 06	** − **7.5214	0.000***	** − **1.41E** − **05	** − **6.135	0.000***	** − **1.62E** − **05	** − **7.672	0.000***
TD	5.19E − 05	4.199	0.000***	5.95E − 05	5.382	0.000***	0.0004	4.170	0.000***	0.0005	5.698	0.000***
Model summary	Adjusted R Square	0.557		Adjusted R Square	0.307		Adjusted R Square	0.508		Adjusted R Square	0.217	
	F	5.156		F	24.210		F	4.405		F	15.536	
	Sig	0.000		Sig	0.000		Sig	0.000		Sig	0.000	
Hausman test	Chi sq. 44.539	Prob	0.000				Chi sq. 34.759	Prob	0.000			

Model	Model 5						Model 6					
	Impact of NPL on ROA by moderating the COVID-19 for IBs Fixed effect			Impact of NPL on ROA by moderating the COVID-19 for IBs Random effect			Impact of NPL on ROE by moderating the COVID-19 for IBs Fixed effect			Impact of NPL on ROE by moderating the COVID-19 for IBs Random effect		
	Coefficients	T	Sig	Coefficients	T	Sig	Coefficients	T	Sig	Coefficients	T	Sig
Constant	− 1.89	− 1.374	.174	− 0.166	− 0.162	.871	− 10.86	− 1.194	.237	5.552	0.787	.433
G.NPL %	− .137	− 5.236	.000***	− .154	− 7.297	.000***	− .435	− 2.509	.015**	− .628	− 4.342	.000***
SIZ	0.525	4.836	.000***	0.377	4.696	.000***	4.349	6.059	.000***	3.006	5.406	.000***
LD %	− .008	− 1.386	.171	− .008	− 2.022	.046**	− .136	− 3.731	.000***	− .121	− 4.267	.000***
EA %	− 0.040	− 0.805	.424	− .026	− 0.886	.377	− 0.513	− 1.549	.127	− .397	− 1.921	.058*
CA %	.066	1.842	.070*	0.031	1.269	.208	.419	1.769	.082*	0.038	0.225	.822
LLR %	− 0.0005	− .481	.632	− 0.002	− 2.129	.036**	.015	2.092	.041**	.003	0.451	.653
LA %	− 0.041	− 3.580	.000***	− 0.031	− 3.572	.000***	− .391	− 5.097	.000***	− .290	− 4.836	.000***
INF	.064	2.179	.033**	0.051	2.107	.038**	.817	4.197	.000***	.770	4.646	.000***
UNE	− 0.053	− 1.908	.061*	− 0.020	− 1.048	.297	− 0.002	− 0.009	.992	.062	0.463	.645
TC	− 1.28E − 06	− 2.867	.006**	− 1.13E − 06	− 2.868	.005**	− 1.54E − 05	− 5.202	.000***	− 1.29E − 05	− 4.828	.000***
TD	7.26E − 05	3.644	.000***	5.06E − 05	3.045	.003**	0.0007	5.386	.000***	− .0005	4.551	.000***
Model summary	Adjusted R Square	0.815		Adjusted R Square	0.698		Adjusted R Square	0.489		Adjusted R Square	0.690	
	F	10.310		F	23.75		F	12.971		F	22.907	
	Sig	0.000		Sig	0.000		Sig	0.000		Sig	0.000	
Hausman test	Chi sq. 15.093	Prob	0.178				Chi sq. 20.375	Prob	0,060			

*G.NPL* gross NPL ratio %; *SIZ* firm size based on log of total assets; *LD* loan-to-deposit ratio%; *EA* equity-to-assets ratio %; *CA* capital adequacy ratio total; *LLR* loan loss reserves to gross NPLs %; *LA* liquid assets to total deposits and borrowings%; *INF* inflation % CPI; *UNE* unemployment, total (% of total labor force) (modeled ILO estimate); *TC* total cases of COVID and *TD* total deaths of COVID

### Robustness analysis

We apply a robustness analysis to rich our findings as presented in Table 10. While we measured the consequence of NPLs over the FP in the original analysis, as a robustness analysis, we measure the reverse association by testing the impact of FP over NPLs during the COVID-19. Khan et al. [56] display that the FP has a significant negative influence over NPLs. This negative link was sustained by Makri et al. [69]. These studies determined that when the FP declines, bank starts to invest in high-risk projects, and then, NPLs upsurge. Rachman et al. [87] study several banking factors that affected the NPLs and find that high profitability of banks has poorer NPLs as a result of their better proceeding activity and operative credit supervision system. While Kumar and Kishore [62] show that FP has insignificant relationship with NPLs. Based on Islamic banking context, Kabir et al. [52] claim that an upsurge in IBs profitability is expected to result in lower CR. Consequently, we suppose that growing of FP generally and during a crisis like COVID-19 has effects on reducing the level of CR.

**Table 10:** Impact of FP as one of the determinants over the NPLs

Model	Impact of FP as one of the determinants over the NPLs for the whole sample			Impact of FP as one of the determinants over the NPLs for the IBs			Impact of FP as one of the determinants over the NPLs for the CBs		
	Coefficients	T	Sig	Coefficients	T	Sig	Coefficients	T	Sig
(Constant)	13.232	3.583	.000***	29.889	3.391	.001**	13.232	3.583	.000***
ROA %	1.422	1.862	.063*	1.230	.661	.512	1.422	1.862	.063*
ROE %	.278	2.926	.004**	− .306	− 1.615	.112	.278	2.926	.004**
SIZ	− 1.146	− 4.543	.000***	− 1.004	− 1.717	.092*	− 1.146	− 4.543	.000***
LD %	− .005	− .382	.703	− .092	− 3.731	.000***	− .005	− .382	.703
CI %	.107	4.996	.000***	− .017	− .462	.646	.107	4.996	.000***
NII %	− .052	− 1.775	.077*	− .004	− .134	.894	− .052	− 1.775	.077*
EA %	− .004	− .036	.971	− .169	− .886	.380	− .004	− .036	.971
TIRE	− .361	− 1.631	.104	− .217	− .959	.342	− .361	− 1.631	.104
CA %	.340	1.533	.126	.142	.639	.525	.340	1.533	.126
LLR %	− .017	− 3.695	.000***	− .011	− 2.750	.008**	− .017	− 3.695	.000***
LA %	.130	6.786	.000***	.009	.206	.838	.130	6.786	.000***
TC	8.468E − 6	1.540	.124	6.987E − 6	1.065	.292	8.468E − 6	1.540	.124
TD	.000	− .591	.555	.000	− .696	.489	.000	− .591	.555
INF	.038	.796	.427	.015	.098	.922	.038	.796	.427
UNE	− .228	− 4.508	.000***	− .274	− 2.934	.005**	− .228	− 4.508	.000***
Model summary	Adjusted R Square	0.388		Adjusted R Square	0.472		Adjusted R Square	0.388	
	F	18.92		F	5.049		F	18.92	
	1			1			1		
	Sig	0.000		Sig	0.000		Sig	0.0000	

*ROA* return on equity %; *ROE* return on assets %; *G.NPL* gross NPL ratio %,; *SIZ* firm size based on log of total assets; *LD* loan to deposit ratio%; *CI* cost-to-income ratio%; *NII* Net interest income to total assets %; *EA* equity-to-assets ratio %; *TIRE* capital adequacy ratio % Tire 1; *CA* capital adequacy ratio total; *LLR* loan loss reserves to gross NPLs %; *LA* liquid assets to total deposits and borrowings%; *INF* inflation % CPI; *UNE* unemployment (% of total labor force); *TC* total cases of COVID and *TD* total deaths of COVID

Based on Table 10, the result supports a positive impact for ROA and ROE on NPLs for the whole sample and CBs while finding insignificant impact for IBs. This result shows to what extent CBs rather than IBs during the current pandemic with high profitability make investments in high-risk projects and reduce the credit supervision system. The insignificant association for IBs shows to what extent IBs are more restricted particularly during the crisis than CBs for their behavior with the NPLs even with the high level of FP. We justify this result based on the unique structures and procedures of these institutions, which switched the traditional system of financing to system that comply with Sharia that is working based on SPL mechanism which makes corporations and investors more restricted in investing during the crisis.

## Conclusion

CR is one of the most significant types of risk faced by financial institutions as banks, and one of the most significant variables affecting their FP. This study seeks to investigate the impact of CR based on NPLs on the FP using empirical evidence from an emerging market (200 banks across MEA region). We covered the panel data from CBs and IBs before and during the COVID-19. The analysis supports for what extent NPLs have a negative impact upon FP. Our analysis reveals that COVID-19 is partially mediated the association between NPLs and FP for CBs. However, we could not approve the same impact across IBs.

As a result, we suggest that banks should change their credit policies to reduce CR, which has an impact on FP. Good credit policies lead to reduced poor credit in banks and improved profitability. For managing CR, banking management should guarantee policies for providing loans and prompt repayment of loan installments timely from borrowers and should monitor the liquidity situation. To meet CR, management should keep a healthy capital charge. For policymaker, to eliminate information asymmetry and hence the likelihood of default, bank management should thoroughly evaluate borrowers' references throughout the credit analysis. As a result, banks must implement powerful credit information systems that assist them in filling informational gaps and increasing access to complete, accurate, and trustworthy data. The regulators must implement an early warning indicator to monitor the accumulation of NPLs to avoid any financial crisis triggered by the presence of NPLs. Islamic Financial Services Board (IFSB) and other IBs regulators as Accounting and Auditing Organization for Islamic Financial Institutions (AAOIFI) should reevaluate policies and products to decrease CR. This study is important to investors, because understanding the relationship between CR and profitability’s will lead to more confident investing behavior. The findings will aid CBs and IBs’ managers in gaining a better understanding of such risks. This will aid in providing insight and understanding into actions aimed at adapting international principles as Basel and unique IBS rules and putting them into effect. The findings would be valuable in creating policy measures to advance the banking industry in MEA region.

The current analysis was limited to the years 2018 to 2020. As a result, future studies may take into account larger data sets in order to conduct more in-depth analysis. Future studies could employ FP based on economic indicator like market capitalization and Tobin's Q. Furthermore, the effects of corporate governance on NPLs, and impact of NPLs on economic development, dividends, cash holdings and stock prices may be considering in the future research. Upcoming research could be conducted in different regions to look into the effects of CR factors and repercussions. An in-depth understanding of how CR has changed FP by the use of a questionnaire and SEM technique, will be an intriguing and instructive study to perform in the future. Because we exclusively focus on developing nations, a comparison of risks with developed countries could be a useful future study. Because this study focuses on financial institutions, future research could compare with non-financial firms. Future research may apply a comparison between the impact of global financial crisis with pandemic of COVID-19 on the link between FP and CR for IBs and CBs.

## Data Availability

The datasets used and/or analyzed during the current study are available from the corresponding author on reasonable request.

## Abbreviations

NPLs: Nonperforming loans
CR: Credit risk
FP: Financial performance
ROA: Return on assets
ROE: Return on equity
IBs: Islamic banks
CBs: Conventional banks
MEA: Middle East and the Africa
OLS: Ordinary least squares
MENA: Middle East and North Africa
GCC: Gulf Cooperation Council
CAR: Capital adequacy ratio
MPT: Modern portfolio theory
USA: United State of America
SMEs: Small- and Mid-size Enterprise
IFSB: Islamic Financial Services Board
AAOIFI: Accounting and Auditing Organization for Islamic Financial Institutions
